# Epilepsy detection based on spatiotemporal feature interaction fusion of EEG signals

**DOI:** 10.3389/fneur.2025.1478718

**Published:** 2026-01-14

**Authors:** Zhencai Xu, Hongfeng Ge, Weiwei Huang, Hongwei Lu

**Affiliations:** 1 Qingdao Engineering Vocational College, Qingdao, China; 2 CRRC Qingdao Sifang Co., Ltd., Qingdao, China; 3 Guanyun People’s Hospital, Lianyungang, China; 4 Southeast University, Nanjing, China

**Keywords:** epilepsy detection, feature fusion, transformer, GAT, spatiotemporal modeling

## Abstract

**Objective:**

In recent years, with the development of machine learning and deep learning technologies, an increasing number of research works have begun using these technologies for automatic seizure detection in EEG signals. However, existing automatic seizure detection algorithms primarily focus on the features of individual EEG channels and pay less attention to the inter-channel relationships. This results in insufficient extraction of spatiotemporal information from multi-channel EEG data, affecting the final seizure detection performance.

**Methods:**

Therefore, this paper proposes an automatic seizure detection method based on the combination of Graph Attention Networks (GAT) and Transformer networks. Specifically, GAT is used as the front end for extracting spatial features, fully leveraging the topological structure of different EEG channels. Meanwhile, the Transformer network is used as the back end to explore temporal relationships and make final decisions based on the states before and after the current moment.

**Results:**

Experiments were conducted on the CHB-MIT and TUH datasets with ten-fold cross-validation. The final seizure detection accuracies on the two datasets were 98.62 and 98.12%, respectively, with the model’s performance surpassing or being comparable to current state-of-the-art models.

**Conclusion:**

The proposed hybrid algorithm combines the advantages of two deep learning models, fully exploring the spatiotemporal correlations between EEG channels. Experiments on public datasets demonstrate the effectiveness of this method, significantly advancing the development of automatic seizure detection.

## 1 Introduction

Epilepsy is a disease caused by abnormal and persistent discharge of neurons in the brain, leading to brain dysfunction and whole-body convulsions. In the daily life of epilepsy patients, sudden seizures can occur, making them prone to serious injury or even death. Currently, there are over 72 million epilepsy patients worldwide, meaning that on average, 3 to 6 out of every 750 people globally may face the risk of seizures at any time (1–3). Accurate and efficient epilepsy detection is crucial for ensuring people’s safety, which has increasingly become a research hotspot.

In clinical medicine, important methods for diagnosing neurological diseases like epilepsy include functional magnetic resonance imaging (fMRI), magnetoencephalography (MEG), and electroencephalography (EEG). Compared to the high cost of fMRI and MEG, EEG is more cost-effective, and its equipment is easier to use and maintain. EEG is crucial for epilepsy detection as it helps doctors diagnose epilepsy and develop treatment plans based on the severity of the condition. During a seizure, EEG can detect abnormal changes in brain signals, which can help doctors determine the type and location of the seizure. Additionally, EEG helps doctors evaluate a patient’s response to medication and decide whether to adjust the treatment plan. Currently, manual analysis of EEG has many limitations, such as being cumbersome, inaccurate, and influenced by individual differences. Therefore, finding an automated epilepsy detection method is of great significance.

In recent years, research on automatic epilepsy detection algorithms has mainly included machine learning and deep learning algorithms. In traditional machine learning techniques, feature and classifier selection is accomplished through trial and error. Developing reliable models requires deep knowledge of signal processing and data mining techniques. These models perform well on limited data. However, with the increasing availability of data, machine learning techniques may underperform. With the development of deep learning, many deep learning-based seizure detection methods have been proposed. These methods, based on neural networks, offer higher accuracy compared to machine learning-based methods. Currently, many researchers and institutions have applied convolutional neural networks (CNN) and LSTM networks to epilepsy detection with good results. However, because EEG signals are collected using electrodes in three-dimensional space, there are hidden spatial relationships between electrodes. Most current deep learning methods do not consider this, leading to insufficient feature extraction and affecting detection results. Therefore, this paper focuses on the study and experimental analysis of spatiotemporal feature learning in EEG.

To address the issues of unutilized spatial features and insufficient feature extraction in current epilepsy EEG signal detection, this study makes the following contributions:

- Modeling the patient’s multi-channel raw EEG signals as a graph structure to deeply describe the spatial features of EEG signals. Pearson correlation is used to calculate the correlation values between different channels, modeling the input samples as a graph structure based on these correlations. Each channel is viewed as a node, the correlation coefficients between different channels are viewed as edge weights, and the original signal values represent node features. The nodes of the graph structure represent the various channels, and the edges represent the correlations between channels.
- Constructing an automatic epilepsy detection model based on Graph Attention Networks (GAT) and Transformer networks. GAT is used as the front end and the Transformer network as the back end. The GAT layer aggregates spatial features between channels, and the output feature map is then sent to the Transformer network for temporal feature extraction and binary epilepsy classification.
- Conducting experiments and performance validation of the model on the CHB-MIT and TUH datasets, fully demonstrating the effectiveness of the proposed method.

This paper follows the following structure: Section 2 discusses the latest research related to this field. In Section 3, we discuss the overall architecture and implementation details of the proposed algorithm. Section 4 provides an in-depth introduction to the datasets used, experimental details, and experimental results of the model. Section 5 presents the conclusion and future work prospects.

## 2 Related works

With the rapid development of deep learning, many deep learning methods have been applied to EEG analysis and epilepsy detection (4–9). End-to-end deep learning models can eliminate the reliance on manual feature selection. Additionally, deep learning has the advantage of handling multi-channel data. Gramacki et al. proposed an automatic seizure detection method based on Convolutional Neural Networks (CNN), which is an end-to-end learning approach (10). In the work of Sang et al., a one-dimensional CNN seizure prediction method combined with a channel selection strategy was proposed (11). Nogay et al. explained the concepts of pre-trained deep two-dimensional CNN and transfer learning, and proposed an end-to-end machine learning model for seizure detection. Utilizing the architecture of convolutional neural networks improves the average rate of feature extraction and enhances detection accuracy. Deep networks can separate more EEG features. However, since this method relies on the selection of convolution kernels, it is difficult to extract temporal features. Extracting temporal features requires very deep networks, increasing the complexity and computational load of the network structure. Additionally, CNN-based methods may lose some EEG information in three-dimensional space.

To more efficiently extract temporal features, Shekokar et al. proposed an automatic epilepsy detection method based on LSTM (12). LSTM, as a special recurrent neural network structure, is particularly suitable for processing long sequences. Since EEG is a time series, LSTM can be used to extract temporal features without causing gradient explosion or vanishing gradient problems. Using LSTM-based models for automatic epilepsy detection further improves the accuracy, sensitivity, and specificity of experiments. LSTM addresses the gradient explosion problem of Recurrent Neural Networks (RNN), but can only establish unidirectional time series models. Bi-LSTM networks not only solve the gradient explosion problem of RNNs but can also transmit information bidirectionally, making them very suitable for analyzing long sequences. In Hu’s work, an epilepsy detection method based on Bi-LSTM networks was developed (13). Information transmitted from both forward and backward directions was utilized, achieving high accuracy in seizure detection. Additionally, the Gated Recurrent Unit (GRU) model, due to its simplicity, has been proposed for seizure detection (14). However, RNN-based models assign weights to features at the initial stage, making each feature’s impact on the decision the same, which leads to lower accuracy in some methods.

Vaswani et al. proposed the Transformer, which is entirely composed of attention mechanisms (15). The introduction of the Transformer has made significant contributions to the field of deep learning, breaking the previous notion that deep learning models must be sequential inputs. The introduction of the attention mechanism into natural language processing has achieved great success. The Transformer not only performs well in machine translation tasks but has also been successfully applied to language modeling, text classification, speech recognition, brain-computer interfaces, and other fields. The introduction of the Transformer has also provided new ideas for the interpretability and visualization of deep learning models. By visualizing the attention weight matrix, one can intuitively understand which parts of the input the model focuses on, thereby improving the model’s interpretability. This approach has been widely applied in deep learning models. Compared to LSTM and other deep learning models, the Transformer has better parallelism and supports multiple processing of long-sequence data. Since EEG is long-sequence data, the Transformer is suitable for extracting the temporal relationships of EEG signals (16).

The aforementioned deep learning methods represent EEG signals from different channels in a 2D manner, which may lead to information loss because the channels are deployed in a 3D space. When processing 3D data, CNNs divide the 3D data into multiple planes in 2D and then perform convolution operations, which may result in information loss. Moreover, the fixed convolution kernel size of CNNs may not fully capture all the information in 3D space, leading to further information loss. In contrast, graph networks can directly process graph data in 3D space and retain information in 3D space. Therefore, some researchers have begun to consider using graph networks to model EEG signals for 3D spatial feature extraction. Hussain et al. proposed a hybrid model for single-patient detection based on CNN and LSTM (17). The combination of CNN and LSTM is effective, and adding convolutional layers to RNNs helps efficiently discover connections in signal channel space (18, 19). However, this still does not solve the problem of information loss between EEG channels in 3D space. In Adeli et al.’s work, a spatiotemporal wavelet-chaos method was proposed to analyze EEG and different sub-bands to detect potential biomarkers of Alzheimer’s disease (20).

To better capture spatial dependencies, several studies have adopted Graph Neural Networks (GNNs), particularly Graph Attention Networks (GAT). For example, Jibon et al. (21) proposed a GAT-based hybrid model that leverages spatial interactions among EEG electrodes for seizure classification. Other works (22, 23) also demonstrated that GAT-based architectures can effectively represent the brain network structure by learning attention-based connectivity graphs. However, these studies mostly focus on spatial modeling, lacking mechanisms to capture complex temporal dynamics. Parallel to this, Transformer-based architectures have recently been introduced to model long-range temporal dependencies in EEG signals. Studies such as (24, 25) used Transformer encoders to capture sequential patterns in EEG for seizure prediction. Nevertheless, most existing approaches, including CNN-based and deep learning frameworks for seizure analysis (35, 36), treat each EEG channel independently and do not explicitly consider the spatial topology or inter-channel relationships.

Apart from deep learning models, biologically inspired models such as NeuCube (26) have been proposed to account for both spatial and temporal aspects of brain activity. NeuCube represents EEG channels as spatially distributed spiking neurons and models temporal information through spatio-temporal spike trains. While effective in theory, NeuCube-based methods often require complex encoding, suffer from scalability issues, and are less compatible with modern deep learning frameworks.

In contrast to these approaches, our proposed model integrates both GAT and Transformer in a unified framework. This design enables simultaneous extraction of spatial features through attention-based graph learning and temporal features via global sequence modeling. By fusing spatiotemporal representations, the model more effectively captures dynamic EEG patterns associated with seizures.

## 3 Methods

This method explores the spatiotemporal features of epileptic EEG signals using GAT and Transformer. The specific process is as follows:

Firstly, the MNE toolbox in Python is used to read the raw EEG data from the public dataset into a format that the computer can recognize. The read EEG data is then divided into shorter time windows. The segmented signal time windows are passed through a bandpass filter and then normalized. The processed EEG data is divided into training and testing sets according to a certain ratio.

For each segment of the divided data, the Pearson correlation is calculated to determine the correlation between channels, ultimately obtaining the graph structure. This maps the signal data of each channel and the hidden spatial relationships between multiple EEG channels to the nodes and edges of the graph, respectively.

**Figure 1:**
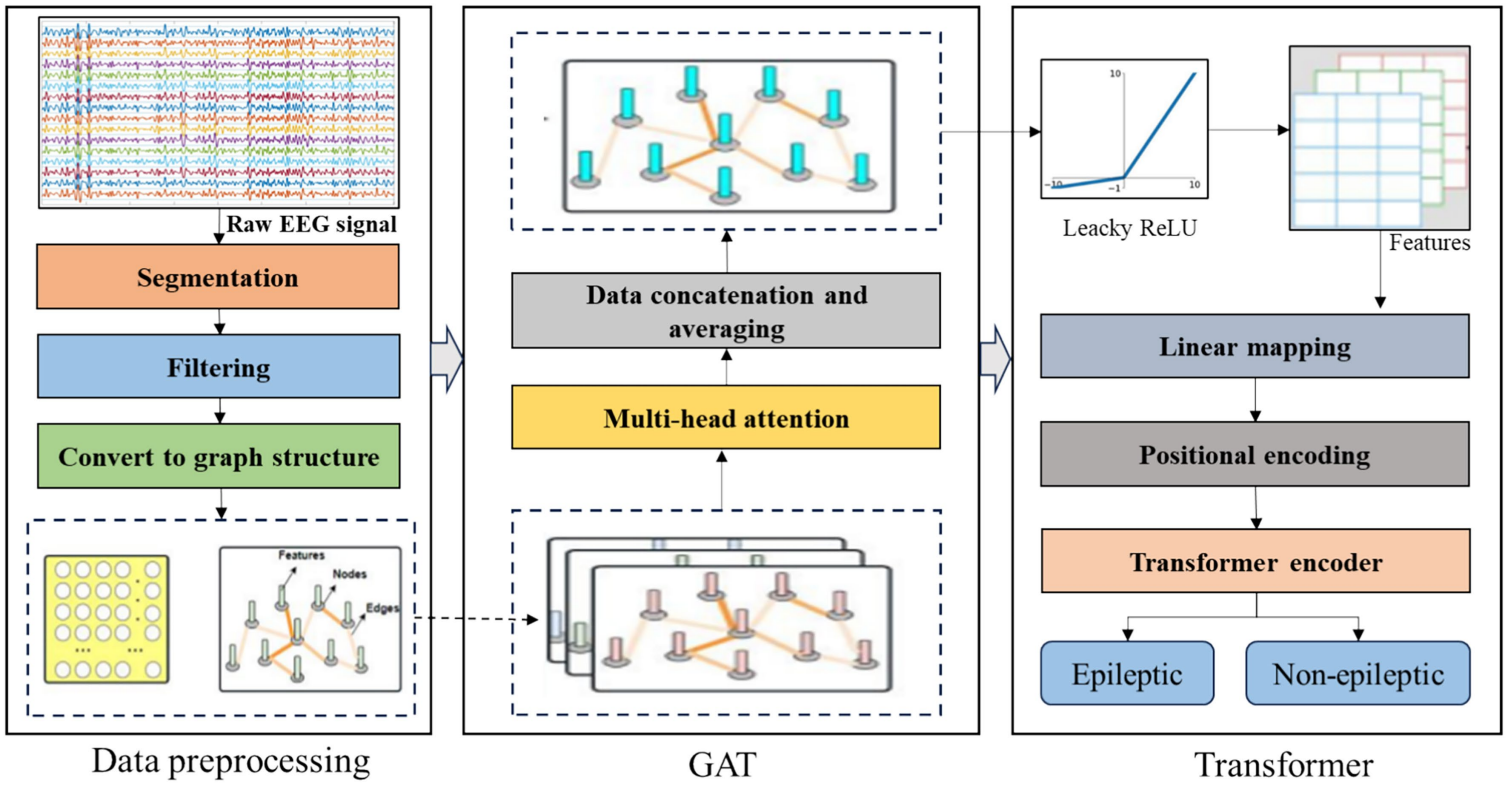
Overall flow diagram of the model.

The preprocessed EEG data and the generated graph structure are input into the GAT model for spatial feature extraction. After computation through the multi-head attention mechanism, a new graph structure is obtained. This operation is repeated once more to finally obtain a new graph structure that aggregates the features of adjacent nodes. This graph structure contains the spatial information between EEG channels. Then, this structure is input as a time series into the Transformer network for further temporal feature extraction. Finally, the output result is fed into a Softmax classifier for binary epilepsy detection. The overall flow of the model is shown in Figure 1.

The detailed mathematical formulations of the proposed method are provided in Equations 1–15.

### 3.1 Graph modeling method

To assess the correlation between different channels of EEG signals, Pearson correlation coefficient is employed to measure the degree of correlation between pairs of channels. Each channel is treated as a node, with the correlation coefficient between different channels representing the edge weight. The original values of the signals serve as node features. Pearson correlation coefficient is a commonly used statistical method to quantify the correlation between two variables. It ranges from −1 to 1, where stronger correlations approach 1 or −1. In the field of seizure detection, Pearson correlation is widely used to assess the correlation between EEG signal features and seizure occurrences. In this paper, Pearson correlation is used to compute the correlation coefficients between different EEG channels.

The Pearson correlation coefficient, which measures the degree of correlation between two variables, can be calculated using the formula below:


Person=C(X,Y)/(S(X)∗S(Y)) (1)

**Figure 2:**
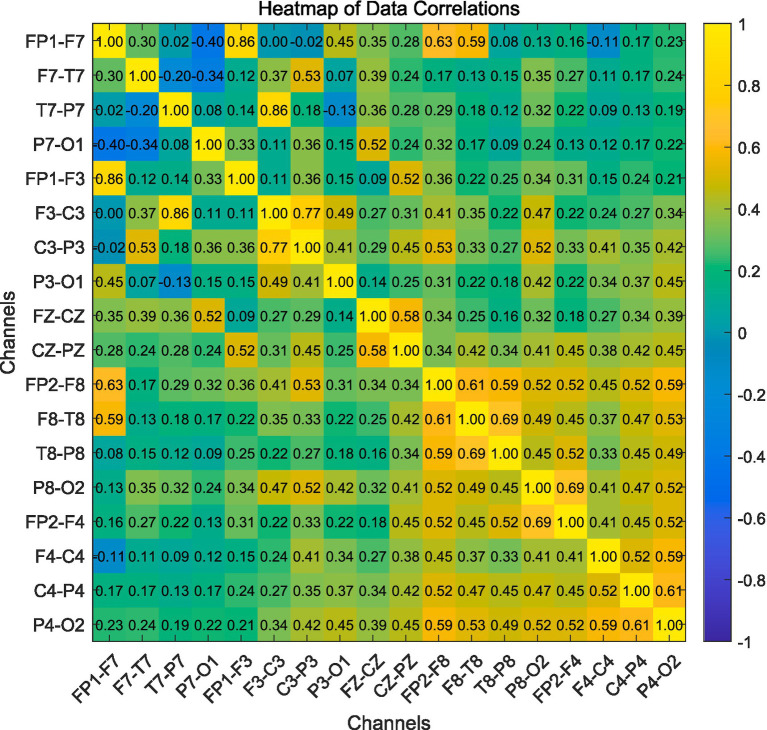
Correlation matrix.

Where X and Y are two variables, C(X, Y) represents the covariance between X and Y, S(X) denotes the standard deviation of X, and S(Y) denotes the standard deviation of Y. Typically, the strength of the correlation between variables is judged based on the following ranges of values:

Using computed Pearson correlation coefficients to form a correlation matrix. A correlation matrix is used to represent the relationships between multiple variables. It is an n*n matrix where n is the number of variables, and each element corresponds to the correlation between each pair of variables. After obtaining the correlation matrix, as shown in Figure 2, a threshold is set. If the correlation between channels is less than this threshold, the corresponding value in the correlation matrix is set to 0; if it exceeds the threshold, it is set to 1. Finally, edges are formed in the graph based on the non-zero values in the matrix, thereby constructing the graph structure.

In this study, the EEG signals are recorded and processed using a bipolar montage configuration, as defined in the CHB-MIT dataset. In this setup, each EEG channel represents the voltage difference between two electrodes placed on the scalp. For example, the label “Fp1–F7” denotes the differential signal between electrodes Fp1 and F7, rather than a single electrode or location. This montage is commonly used in clinical EEG recordings to enhance local signal contrast and reduce common-mode noise. Throughout this paper, we adopt the standard bipolar montage naming convention used in the dataset and ensure that all references to channels accurately reflect this configuration.

Figure 2 shows the correlation matrix of 16 EEG channels for a given subject. It reveals strong correlations between several channel pairs, such as ‘FP1-F3’ and ‘FP1-F7’, ‘P7-O1’ and ‘P3-O1’, ‘P6-O1’ and ‘P4-O2’, ‘P3-O2’ and ‘P3-O4’, and ‘FP2-F4’ and ‘FP2-F8’, with correlation coefficients of 0.86, 0.69, 0.71, 0.77, and 0.76, respectively. While some of these strongly correlated channels are spatially adjacent, others are not, indicating that Pearson correlation captures functional rather than purely anatomical relationships. Therefore, in our approach, EEG signals are modeled as a graph based on functional connectivity. Each EEG channel is treated as a node, and the edges between nodes are determined by thresholded Pearson correlation coefficients, representing the statistical interactions between signals. Each node stores the original EEG signal of the corresponding channel. This graph is then input into a Graph Attention Network (GAT), which applies a self-attention mechanism to dynamically evaluate the importance of neighboring nodes. By using functional correlations as edge weights, GAT enables fine-grained modeling and extraction of spatial features from EEG data, thereby facilitating accurate and efficient detection of epileptic seizures.

### 3.2 Graph attention network

Graph Attention Networks (GAT) are a type of graph neural network model based on attention mechanisms, used for processing graph data. Research on GAT began in 2017 and has since been widely applied in natural language processing, recommendation systems, bioinformatics, and other fields (22). The core idea of GAT is to apply attention mechanisms on each node in the graph to autonomously learn important relationships between nodes. This allows GAT to better capture complex structural information within the graph, outperforming traditional graph neural network models.

In recent years, GAT has garnered significant attention and achieved notable results in the fields of graph data processing and biomedical domains. For example, GAT has been successfully applied to tasks in natural language processing, achieving state-of-the-art results. In the domain of EEG signals, GAT has also been extensively utilized. For instance, GAT can be employed for classification and analysis of EEG signals to detect epileptic activities or other anomalies.

In EEG signal analysis, GAT can extract spatial features from EEG graphs. GAT learns the interdependencies between different leads in EEG signals and utilizes these relationships for classification or other analyses. Compared to traditional graph neural network models, GAT exhibits strong learning capabilities and generalization, enabling it to capture complex structural information within EEG graphs. Therefore, research on GAT in the EEG domain has received widespread attention and achieved significant advancements. In the field of epilepsy detection, GAT is widely used in research to detect epileptic activities with high accuracy and specificity. Next, we will detail the principles of the Graph Attention Layer and its application in epilepsy detection experiments.

The input to the GAT module is a set of feature vectors for nodes, and the output is a set of new feature vectors for nodes. To enhance the representativeness of learned features, a shared weight matrix is applied for linear mapping on each node. The transformed features are then concatenated, and the concatenated high-level feature mapping is mapped to real numbers. The formula can be expressed as:


$$k=\{k_{1},,,k_{2},,,\ldots ,,,k_{M}\},k_{i}\in R^{F}$$ (2)

$$e_{\mathrm{ij}}=g(Wk_{i},Wk_{j})$$ (3)

Here, *W* represents the trainable weight matrix, *e* denotes the correlation coefficient between two nodes, *k* represents the feature vector of nodes, and *g* represents the linear mapping. Next, we need to compute the weights between nodes. Typically, attention mechanisms distribute attention across all nodes in the graph, potentially resulting in loss of structural information. Here, we only consider the structure formed by the first-order neighboring nodes of the nodes. The weight calculation is as follows:


$$\alpha_{\mathrm{ij}}=\text{soft}\max_{j}(e_{\mathrm{ij}})=\frac{\exp(e_{\mathrm{ij}})}{\sum_{r\in V_{i}}\exp(e_{\mathrm{ir}})}$$ (4)

Where *α* represents the weights between nodes, and *V* represents the set of first-order neighboring nodes of the nodes. Using the LeakyReLU activation function, the fully expanded formula can be expressed as:


$$\alpha_{\mathrm{ij}}=\frac{\exp(\text{LeakyReLU}(a^{T}[{\mathrm{Wk}}_{j}{\mathrm{Wk}}_{j}^{\prime }]))}{\sum_{r\in V_{i}}\exp(\text{LeakyReLU}(a^{T}[{\mathrm{Wk}}_{j}{\mathrm{Wk}}_{j}^{\prime }])}$$ (5)

Where $a^{T}\in R^{2F}$ is the parameter of the feedforward neural network, LeakyReLU is the non-linear activation function, ∣∣ denotes concatenation operation. To enhance the model’s fitting capability, this paper employs multi-head attention mechanism. To ensure the stability of the GAT model, the results of multiple attention mechanisms are averaged, and each attention mechanism has different learning characteristics. Therefore, the computation of learned features for nodes is as follows:


$$k_{i}^{\prime }=\sigma (\frac{1}{q}\sum_{q=1}^{Q}\sum_{j\in V_{i}}\alpha_{\mathrm{ij}}^{q}W^{q}k_{j})$$ (6)

Where *q* represents any one of the heads in the multi-head attention mechanism, and $k_{i}^{\prime }$ represents the output aggregated from neighboring node information.

**Figure 3:**
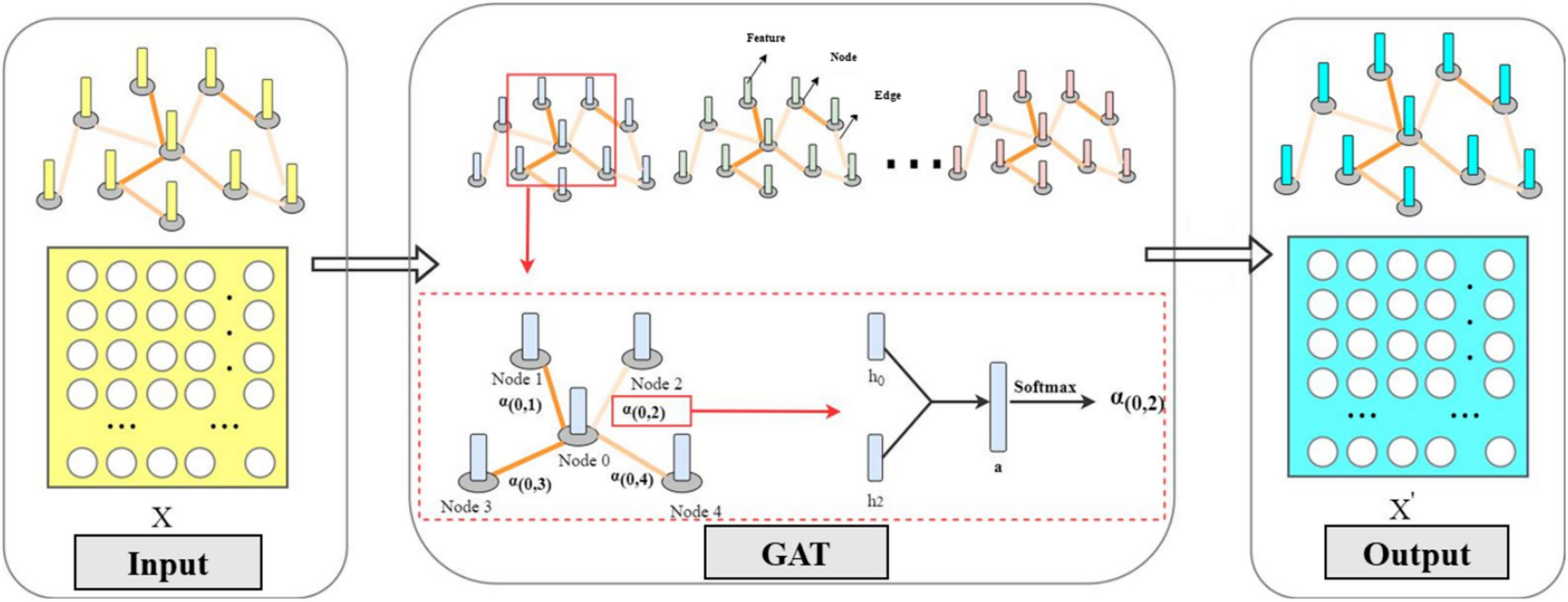
Graph attention layer calculation details.

Figure 3 illustrates the detailed computational process of the graph attention layer, where each patient is modeled as a graph structure. Spatial correlation aggregation is then performed to compute attention coefficients for each node and its neighbors, aggregating spatial correlations across channels. Finally, the output of the multi-head attention mechanism is obtained, which constitutes a feature vector set aggregating spatial correlations between nodes. With the introduction of attention mechanisms, weights are shared only with adjacent nodes, without requiring information from the entire graph. If the connection between two nodes is missing, their attention coefficients are not computed. Therefore, this model exhibits robustness to interference and can effectively extract spatial relationships between channels.

### 3.3 Transformer network

Compared to traditional recurrent neural networks like Bi-LSTM, Transformer offers several advantages, especially in handling long sequences. Firstly, Transformer utilizes attention mechanisms, enabling better learning of long-term dependencies. Secondly, its structure is simpler as it does not require recurrent connections or hidden states to retain information, allowing for faster training and decision-making. These advantages make Transformer particularly suitable for tasks involving learning from long sequences. In the field of EEG signal analysis, Transformer networks are employed to detect abnormal activities in EEG signals of epilepsy patients. Leveraging its strong pattern recognition capabilities, Transformer models can identify complex patterns within time series data. In epilepsy detection, Transformers are used to recognize electroencephalographic signals preceding seizures, facilitating early diagnosis and prevention. Additionally, Transformers classify different types of seizures and assess the severity of epilepsy in patients.

Overall, research in applying Transformers to EEG signals and epilepsy detection has made significant strides. Its capability to effectively handle variable-length sequences and high-dimensional data underscores its critical role in early diagnosis and prevention of epileptic seizures. Next, we will delve into the principles of Transformer networks and their applications in epilepsy detection experiments.

We use Transformer to extract the temporal information of EEG. The output of GAT is linearly encoded and mapped. Then, before entering the Transformer encoder, positional embeddings are added to the flattened 2D slice elements. Finally, softmax function completes the classification.

To match the input with the Transformer encoder, the input $x\prime \in R^{M\times F\prime }$ is split into a series of fixed-size μ1×μ2 flattened 2D slices. Thus, each slice represents 1 s of multi-channel EEG signal. Then, all slices are mapped to higher dimensions for further training through a linear projection layer. Additionally, to obtain global correlations, a class label *X’* is added, which is also an element used for the final classification task. The Transformer model processes sequential data as a whole. To make the model understand positional information, positional encoding vectors are added to the input embeddings. The core idea is to provide practical distance information in attention computation.

The values of encoding vectors usually follow specific patterns. In this work, sine and cosine functions are chosen as encoding vectors. Based on this, standard one-dimensional positional embeddings $\text{EPOS}\in R^{(N+1)\times D}$ are added to the slice embeddings. The final input to the Transformer encoder is:


$$H=[k_{\text{class}}^{\prime },E\cdot {k_{\mu }^{\prime }}^{1},E\cdot {k_{\mu }^{\prime }}^{2},\ldots ,E\cdot {k_{\mu }^{\prime }}^{N}]+{E_{\mathrm{pos}}}^{'}$$ (7)

**Figure 4:**
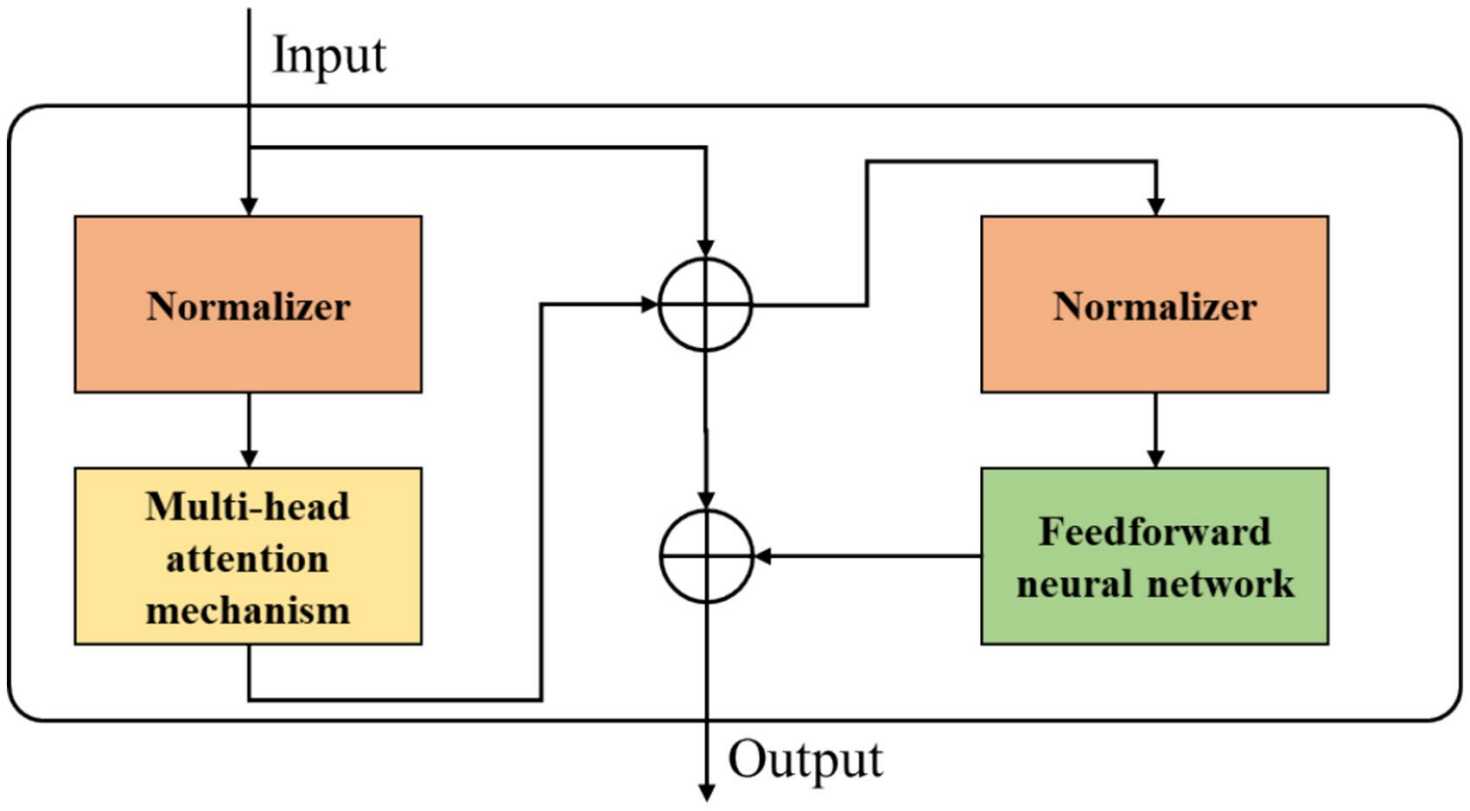
The specific structure of the Transformer encoder.

We attempts to encode input vectors through encoder blocks. The Transformer architecture consists of L stacked encoder modules. Each layer comprises two sub-modules: a multi-head self-attention mechanism and a fully connected feed-forward network, structured as shown in Figure 4. Residual networks are used for each Transformer encoder to retain input feature information and enhance model stability. The input of the Transformer encoder is first processed by the self-attention mechanism. During computation, multi-head attention layers are used to enhance the performance of the self-attention layer. Then, feature vectors are further embedded through a feed-forward neural network. The specific calculation process is as follows:


$$H_{l}^{\prime }=\mathrm{MSA}(\mathrm{LN}(H_{l-1}))+H_{l-1},l=1,2,\ldots ,L$$ (8)

$$H_{l}=\mathrm{MLP}(\mathrm{LN}(H_{l}^{\prime }))+H_{l}^{\prime },l=1,2,\ldots ,L$$ (9)

In this context, $H_{l-1}$ represents the input to encoder *l*, and $H_{l}$ represents the output of the encoder. In this work, *L* is set to 4.

### 3.4 Loss function

In epilepsy detection, class imbalance between positive and negative samples is a common issue. When the numbers of positive and negative samples are imbalanced, the model tends to bias toward the majority class, leading to poorer recognition of the minority class. To address the imbalance in EEG-based seizure detection, a focal loss function has been introduced to the model. The focal loss function is designed to optimize classifier performance by adjusting the classifier’s focus during training.

In the context of epilepsy detection research, the focal loss function has been used to enhance the performance of EEG classifiers. By assigning larger weights to minority class samples and smaller weights to majority class samples in the loss function, the focal loss function allows the model to pay more attention to minority class samples. This approach enhances sensitivity and specificity in epilepsy detection algorithms by prioritizing accuracy in classification.

In this chapter’s epilepsy detection task, training the classifier with imbalanced samples, the use of focal loss function can address the issue of sample imbalance, which better aligns with clinical medical practices.

The focal loss function modifies the cross-entropy loss function by reducing the weight of the majority of samples during training. The binary cross-entropy loss function is given by:


$$\mathrm{CE}=\{\begin{array}{l} -\log p,y=1\\ -\log(1-p),y=0 \end{array}$$ (10)

In this equation, *y* represents the true label, and *p* is the output of the activation function (such as the sigmoid function), which ranges from 0 to 1. In this scenario, the loss function will dominate the direction of gradient descent but may overshadow the influence of minority class samples due to sample imbalance.


$$\mathrm{FL}=\{\begin{array}{l} -\alpha {\left(1-p\right)}^{\gamma }\log p,y=1\\ -(1-\alpha )p^{\gamma }\log(1-p),y=0 \end{array}$$ (11)

*α* balances the importance of majority and minority samples, while *γ* adjusts the reduction rate. When γ = 0, the cross-entropy loss function and the focal loss function are equivalent. γ > 0 reduces the loss of easily classified samples, making the model pay more attention to hard-to-classify samples. As γ increases, the influence of the adjustment factor also increases.

## 4 Experiment

### 4.1 Datasets

#### 4.1.1 The CHB-MIT dataset

The CHB-MIT dataset is a public dataset used for epilepsy detection and diagnosis, jointly established by the Massachusetts Institute of Technology (MIT) and the National Institutes of Health (NIH) in the United States [38]. The dataset includes EEG data from 23 patients with intractable epilepsy, divided into 24 recordings as shown in Tables 1, 2. Each case comprises 9 to 42 continuous edf files from individual subjects, with each patient having two or more hours of recordings. It includes known epilepsy patients and non-epilepsy subjects as controls. The recordings are derived from EEG signals from 18 electrodes, including 6 frontal electrodes, 6 top electrodes, and 6 temporal electrodes. All signals are sampled at a resolution of 16 bits with a frequency of 256 Hz. The CHB-MIT dataset captures long-term multi-channel EEG recordings of children with refractory epilepsy and can be accessed via PhysioNet.org. The recordings adhere to the international standard 10–20 system. The total duration of these EEG signal files amounts to 979 h.

**Table 1:** Correlation coefficient and correlation degree correspondence table.

Correlation coefficient	Correlation
0.8–1.0	Very strong correlation
0.6–0.8	Strong correlation
0.4–0.6	Moderate correlation
0.2–0.4	Weak correlation
0.0–0.2	Very weak correlation or no correlation

**Table 2:** Comparison of results of ablation experiments.

Model	Accuracy (%)	Sensitivity (%)	Specificity (%)	Dataset
GAT	96.28	93.03	92.75	CHB-MIT
Transformer	94.16	96.65	94.34	CHB-MIT
GAT+Transformer	98.41	97.73	96.84	CHB-MIT
GAT	95.50	94.95	96.52	TUH
Transformer	98.02	97.70	99.06	TUH
GAT+Transformer	98.12	98.10	99.42	TUH

Due to the variability in EEG electrodes collected from each patient, this paper selects a set of 16 common channels from 16 patients. In the CHB-MIT dataset, we chose the following 16 channels: “FP 1- F 7”, “F7- T7”, “T7 -P 7”, “P7- O1”, “FP 1- F 3”, “F3 -C 3”, “C3 -P 3”, “P3 -O 1”, “FP 2- F 4”, “F4- C 4”, “C4 -P 4”, “P4 -O 2”, “F8 -T8”, “FZ -C Z”, “CZ -P Z”, “FP 2- F 8”.

In the CHB-MIT dataset, EEG is composed of long-term signals, which are segmented into smaller slices. The long-term EEG signals are divided into small segments using a sliding window. Each window lasts for 1 s, with an overlap rate of 0.5. For example, given a 60-s EEG signal with a sampling frequency of 1 Hz, the dimension of the EEG signal is 60. After data segmentation, there will be 119 segments. Through the proposed method, these segments are analyzed and labeled in continuous sequence. Single-subject experiments are conducted with 3,000 s of signal for each patient. To ensure sample balance, the positive and negative ratio is set to 1:1. Therefore, the duration of seizure and normal signals is 1,500 s each. If the duration of a patient’s seizure signal is less than 1,500 s, oversampling is used. Additionally, ten-fold cross-validation is employed to ensure the stability of the experiments.

#### 4.1.2 The TUH dataset

The TUH dataset is a large-scale multimodal EEG database, including long-term EEG recordings and associated clinical information from over 20,000 patient cases. Created by the Medical Center and Neuroscience Research Institute at Temple University in the United States, it aims to facilitate neuroscience research and the development of EEG signal processing algorithms [39]. The dataset comprises extensive EEG data collected using advanced medical equipment, encompassing routine EEGs, long-term EEGs, and continuous EEGs. Clinical information related to EEGs, such as patient medical histories, diagnoses, and treatment plans, is also included. The TUH dataset is a highly valuable resource for studying EEG and magnetoencephalography data, applicable in various research areas including epilepsy detection, stroke diagnosis, brain injury treatment, etc. Moreover, the TUH database is publicly accessible, allowing free access to its contents. It contains over 25,000 EEGs, along with neurologist diagnostic records, patient histories, gender, and age information. EEG data is stored in edf file format, which preserves essential metadata.

The TUSZ dataset is a subset of the TUH dataset, containing EEG data from 593 epilepsy patients with a total duration of 393.58 h. The patients’ ages range from 1 month to 83 years, with approximately equal gender distribution. Each patient has multiple EEG recordings varying in length from minutes to hours. The EEG data differs in terms of channel count, sampling length, and electrode layout. The original signal is sampled at 250 Hz, with each recording having between 20 to 128 channels. The TUSZ dataset provides rich clinical annotation information, including basic patient information, epilepsy types, medication details, medical images, surgical records, etc. These annotations make the TUSZ dataset an ideal resource for automated epilepsy detection and classification. The main directory of the TUSZ dataset includes a CSV file containing metadata about each patient and their samples, such as patient ID, sample ID, sample duration, sample type (normal or epilepsy), etc. Each patient in the TUSZ dataset has a separate folder named after their patient ID. Within each patient’s folder, there is a subfolder named “seizures” containing data files for all epilepsy samples, and a subfolder named “no_seizures” containing data files for all normal samples. Each data file is in edf format, containing one or more records of EEG signals along with metadata such as sampling frequency and channel count. The experiment is based on version 1.5.1 of TUH and the dataset is currently undergoing continuous updates.

In our work, we utilized the TUH dataset for validating the stability of our model. We selected 20 electrodes from the TUH dataset, including “FP1-F7,” “F7-T3,” “T3-T5,” “T5-O1,” “FP2-F8,” “F8-T4,” “T4-T6,” “T6-O2,” “T3-C3,” “C3-CZ,” “CZ-C4,” “C4-T4,” “FP1-F3,” “F3-C3,” “C3-P3,” “P3-O1,” “FP2-F4,” “F4-C4,” “C4-P4,” and “P4-O2.”For the experiments involving single-subject validation, due to significant variability among patients in the TUH dataset, we selected 10 patients. Each patient’s sample duration was set to 3,000 s, with a positive-to-negative ratio of 1:1. We employed ten-fold cross-validation to ensure the stability of our experimental results, following a similar approach as with the CHB-MIT dataset.

### 4.2 Experimental setup and evaluation metrics

In this experiment, a one-second sliding window was used to analyze EEG records from different channels. Due to the scarcity of seizure samples in the dataset, we employed an overlapping window method with a overlap rate of 0.5 to segment seizure data. The test data was normalized using the mean and variance from historical training data of the same patient.

Furthermore, the proposed GAT-Transformer model was implemented in PyTorch and optimized using the Adam optimizer with an initial learning rate of 0.001. The model was trained for 100 epochs with a batch size of 64. To improve generalization, a dropout rate of 0.5 was applied after each layer. The GAT module consisted of two layers, each with 8 attention heads. The focal loss function was adopted with *γ* = 2 and *α* = 0.25 to address class imbalance. All experiments were conducted on an NVIDIA GPU server.

The evaluation metrics chosen for this paper include: accuracy, sensitivity, specificity, F1 score, and AUC. The specific calculation methods are as follows:

(1) Accuracy: The ratio of correctly predicted samples to the total number of samples.


$$\text{Accuracy}=\frac{\mathrm{TN}+\mathrm{TP}}{\mathrm{TN}+\mathrm{FP}+\mathrm{TP}+\mathrm{FN}}$$ (12)

(2) Sensitivity: Sensitivity represents the proportion of positive cases that are correctly identified as positive. Numerically, it is equal to the recall rate. It indicates the sensitivity of a detection method to the disease. A high sensitivity detection method means it can detect the disease as much as possible without missing diagnoses.


$$\text{Sensitivity}=\frac{\mathrm{TP}}{\mathrm{TP}+\mathrm{FN}}$$ (13)

(3) Specificity: Specificity represents the proportion of negative cases that are correctly identified as negative. It indicates the specificity of a detection method for non-disease cases. A high specificity detection method means it can correctly diagnose healthy individuals as negative as much as possible, without falsely diagnosing them as diseased.


$$\text{Specificity}=\frac{\mathrm{TN}}{\mathrm{TN}+\mathrm{FP}}$$ (14)

(4) F1 Score: The F1 score is the harmonic mean of precision and recall. It reflects the balance between accurately predicting positive samples and recalling all positive samples. A higher F1 score indicates that the model can accurately predict positive samples while recalling as many positive samples as possible. In the field of epilepsy detection, the F1 score is an important evaluation metric because epilepsy is a serious disease that needs to be detected as much as possible while minimizing misdiagnoses.


$$F1\;\text{Score}=\frac{\frac{\mathrm{TP}}{\mathrm{TP}+\mathrm{FP}}*\frac{\mathrm{TP}}{\mathrm{TP}+\mathrm{FN}}}{\frac{\mathrm{TP}}{\mathrm{TP}+\mathrm{FP}}+\frac{\mathrm{TP}}{\mathrm{TP}+\mathrm{FN}}}$$ (15)

(5) AUC: The Area Under the ROC Curve (AUC) reflects the trade-off between true positive rate and false positive rate in model predictions, used to measure binary classification models.

In the above evaluation metrics: TP (True Positive) represents the number of correctly detected epileptic seizure segments. FN (False Negative) represents the number of epileptic seizure segments that were incorrectly identified. TN (True Negative) represents the number of correctly detected non-epileptic segments. FP (False Positive) represents the number of non-epileptic segments that were incorrectly detected as epileptic.

### 4.3 Ablation experiment

To validate the contributions of the GAT and Transformer combination in the model, a ablation experiment was conducted comparing the GAT model, Transformer model, and the combined GAT + Transformer model. To ensure fairness in the experiment, the model parameters were set identically, as shown in Table 2.

The combined model showed significant improvements in accuracy, sensitivity, and specificity compared to the individual models. For instance, on the CHB-MIT dataset, the GAT + Transformer model improved by at least two percentage points in accuracy, sensitivity, and specificity compared to using GAT and Transformer separately. Similar results were observed on the TUH dataset.

Comparing with using GAT and Transformer separately, the GAT + Transformer model performed the best, confirming this conclusion across both datasets. Due to its ability to leverage the temporal and spatial relationships between EEG channels effectively, the combined model exhibited significantly enhanced learning capabilities.

### 4.4 Single-patient experiment

The proposed method was further validated through ten-fold cross-validation experiments on both the CHB-MIT and TUH datasets for individual patients. Each patient’s seizure and non-seizure samples were divided into ten folds, ensuring that each fold contained both seizure and non-seizure samples to capture features and changes in both classes during training.

The performance of the GAT + Transformer architecture in seizure detection was evaluated by comparing overall accuracy, sensitivity, specificity, and F1 score. To mitigate overfitting, all experiments were repeated ten times. The average results for all patients are shown in Tables 3, 4.

**Table 3:** Table of results of GAT+Transformer crossover experiments (CHB-MIT dataset).

ID	Accuracy	Sensitivity	Specificity	F1 Score	AUC	*p*-value
1	99.73	99.39	99	99.2	99.44	2.5823e–08
2	100	100	100	100	100	1.3729e–08
3	99.9	99.8	99.6	99.7	99.78	1.8517e–08
4	97.37	90.03	95	92.68	96.44	6.9786e–07
5	98.73	99.38	93	95.77	96.44	8.2401e–06
6	96.8	89.1	92.45	90.74	95.09	3.8514e–04
7	98.33	96.6	96	95.05	97.4	1.1628e–08
8	98.67	98.94	93	95.88	96.4	2.1352e–07
9	99.67	99	99	99	99.5	8.0952e–08
10	99.83	100	99	99.49	99.5	5.5879e–08
11	99.2	100	95.2	97.47	97.6	2.5588e–05
12	94.67	100	88	94	91	7.2723e–08
13	97.47	95.17	88.92	92.02	94.24	2.4360e–06
14	94.87	85.71	82.76	84.21	90.04	1.7909e–04
15	99.83	100	99	99.5	99.5	4.1112e–06
16	99.47	100	96.88	98.41	98.44	6.1636e–05
17	99.5	98.99	98	98.49	98.9	2.7252e–07
18	99.83	100	99	99.5	99.5	8.6932e–08
19	98.44	99.28	91.69	95.05	95.57	4.5992e–08
20	97.83	96.77	90	93.26	94.7	1.0190e–07
21	98.56	98.68	92.6	95.54	96.18	6.8914e–04
22	98.97	99.21	94.57	96.75	97.2	7.1290e–08
23	99.59	100	97.53	98.75	98.76	2.1252e–04
24	97.5	100	84	91.03	92	9.3770e–03
Mean±std	98.52 ± 1.48	97.75 ± 3.91	94.34 ± 4.88	95.9 ± 3.84	96.81 ± 2.83	

**Table 4:** Table of results of GAT+Transformer crossover experiments (TUH dataset).

ID	Accuracy	Sensitivity	Specificity	F1 Score	AUC	*p*-value
00003208	96.5	95.15	98	94.18	94.5	8.4333e-08
00005804	94.8	93.43	99.39	95.76	94.81	2.0450e-06
00002521	99.4	99.2	99.39	99.3	99.1	1.0081e-08
00000302	97.2	97.83	99.8	98.46	98.7	1.1838e-07
00002806	100	100	100	100	100	3.1645e-06
00000002	99.5	100	100	96.04	100	8.5412e-08
00006514	95.2	99.3	99.6	96.9	97.2	5.8956e-07
00001587	95.9	99.2	99.3	96.7	98.4	2.1542e-07
00003636	96.6	96.8	99.1	98.69	98.6	8.0952e-08
00007252	98.6	97.54	100	98.6	97.8	1.5535e-07
Mean±std	98.02 ± 1.74	97.7 ± 3.63	99.46 ± 1.62	97.86 ± 1.89	97.8 ± 2.03	

On the CHB-MIT dataset, the method achieved average accuracy, sensitivity, and specificity of 98.52, 97.75, and 94.34%, respectively. On the TUH dataset, the average accuracy, sensitivity, and specificity were 98.02, 97.70, and 99.06%, respectively. Most patients had sensitivity metrics exceeding 95%, with several achieving 100% sensitivity.

Regarding specificity, 30 patients had specificity values exceeding 90%. Only 4 patients had specificity below 90%, partly due to fewer seizure episodes and significant external noise affecting scalp EEG recordings.

On the CHB-MIT dataset, the F1 score and AUC were 95.9 and 96.81%, respectively, while on the TUH dataset, the F1 score was 97.86% and AUC was 97.80%. These results indicate satisfactory accuracy and stability of the model, assisting clinicians in diagnosis.

Furthermore, all *p*-values were less than 0.005 across all scenarios, indicating significant differences in the decision variables learned by the model, further confirming the effectiveness of the proposed method.

### 4.5 Algorithm comparison and discussion

The above sections present ablation experiments and ten-fold cross-validation experiments on individual patients using Graph Attention Network (GAT) and Transformer networks, followed by analysis. To further validate the model’s performance, comparative experiments with advanced machine learning and deep learning methods are conducted as shown in Table 5. These methods mostly utilize feature extraction and deep learning techniques for seizure detection, showing significant improvements in experimental metrics compared to previous methods.

**Table 5:** Comparison of results with other seizure detection algorithms.

Author	Model	Accuracy (%)	Sensitivity (%)	Specificity (%)
Gill et al. (27)	GMM	86.93	86.26	87.58
Chen et al. (28)	DWT	89.01	88.39	89.62
Jana et al. (29)	1D-CNN	88	88.52	91.11
Tsiouris et al. (30)	LSTM	88.67	89.04	89.52
Zhou et al. (10)	CNN	96.7	95.4	92.46
Hu et al. (31)	Bi-LSTM	93.61	91.69	92
Zhang et al. (32)	Bi-GRU	98.49	89.98	98.49
Janjarsitt (33)	Wavelet+SVM	96.87	72.99	93.13
Hussain et al. (17)	1D-convolutional LSTM	95.75	95.77	95.49
Yuan et al. (34)	WPT + ELM	93.86	95.74	92.22
Jibon et al. (21)	GAT	93.87	94.43	93.29
Li et al. (24)	Transformer	94.6	/	89.8
Kasabov (26)	NeuCube	94.23	93.71	90.23
Ours	GAT+Transformer	98.52	97.75	94.34

However, most of these methods only consider limited spatial relationships between EEG channels. From Table 5, it can be seen that the accuracy and sensitivity of GAT + Transformer are higher than most existing methods. Particularly, our method achieves 5.90% higher sensitivity than methods using Bi-LSTM alone and 2.04% higher sensitivity than methods using CNN. This improvement is attributed to better utilization of temporal and spatial information across EEG channels, whereas other methods may lose information in three-dimensional space.

Zhang et al. applied wavelet transform for preprocessing epileptic EEG signals, calculating relative signal energy in specific frequency bands as features input to a Bi-GRU network, achieving satisfactory seizure detection outcomes. Compared to their model, our model exhibits slightly lower specificity by 4.15%, but superior accuracy and sensitivity by 0.03 and 2.01%, respectively. This suggests that their method benefits from manual feature selection and post-processing of model decisions, whereas ours achieves high detection efficiency without complex signal decomposition and post-processing, while also considering spatial relationships between channels.

We also compared our model with recent machine learning or deep learning-based methods for seizure detection. Although the sensitivity is not as high as the WT + SVM method for seizure detection (98.13% vs. 94.34%), our proposed method shows significant improvements in accuracy and sensitivity (98.52% vs. 96.87, 97.75% vs. 72.99%). Hussain et al. employed 1D-CNN-LSTM for classifying epileptic seizure EEG signals, achieving an accuracy of 95.75%, slightly lower than our method, suggesting insufficient feature extraction in their approach.

While some machine learning-based methods show advantages in accuracy, they often require additional manual feature design and optimal classifier selection. In contrast, our method is an end-to-end framework with minimal intervention, simplifying model design and enhancing seizure detection efficiency.

Overall, the performance and stability of our method are satisfactory, validating the effectiveness of combining GAT and Transformer for seizure detection.

## 5 Conclusion

In this chapter, a spatiotemporal seizure detection model based on the GAT + Transformer architecture is proposed. The GAT model is used to capture relationships between original EEG channels and extract spatial features. These features are then fed into the Transformer model to explore temporal relationships and classify epileptic EEG signals. The model considers information from both past and future time points to jointly determine the decision outcome. By combining the strengths of both models, this approach aims to advance automatic seizure detection technology.

This research conducted experiments on publicly available EEG datasets CHB-MIT and TUH. The performance on both datasets either outperformed existing techniques or was comparable, providing comprehensive validation of the model’s effectiveness in seizure detection.

## Data Availability

The original contributions presented in the study are included in the article/supplementary material, further inquiries can be directed to the corresponding author/s.
